# Acupuncture improves spatial learning and memory impairment caused by herpes simplex virus type-1 in rats through the p38 MAPK/CREB pathway

**DOI:** 10.1186/s12576-024-00941-4

**Published:** 2024-10-03

**Authors:** Hongjiao Jin, Rui Huang, Zhu Li, Mi Liu, Ning Zhao, Haiyan Zhang, Yong Lin

**Affiliations:** The First People’s Hospital of Zunyi (The Third Affiliated Hospital of Zunyi Medical University), No. 98 Fenghuang Road, Huichuan District, Zunyi, 563000 Guizhou Province People’s Republic of China

**Keywords:** Herpes simplex encephalitis, Herpes simplex virus type-1, Acupuncture, p38 MAPK/CREB pathway, Cognitive dysfunction

## Abstract

**Background:**

Acupuncture can improve herpes simplex encephalitis (HSE), but the underlying mechanism is not clear. Therefore, we evaluated the cognitive function and apoptosis in hippocampus caused by herpes simplex virus type-1 (HSV-1) in rats after acupuncture and described the molecular mechanism.

**Methods:**

Sprague–Dawley rats were induced into HSE models by HSV-1 infection. After 3 days, they received acupuncture at the acupoints of *Xuanzhong* (GB39), *Baihui* (GV20), *Shenmen* (HT7), *Shenting* (GV24), and *Sanyinjiao* (SP6), and/or intraperitoneal injection of the p38 MAPK inhibitor SB203580. Morris water maze test was performed on rats. The hippocampus of rats was obtained, and the expression of apoptosis-related genes in the tissues was detected by qRT-PCR. In addition, apoptosis-related proteins and proteins related to the p38 MAPK/CREB pathway in the tissues was detected by western blot.

**Results:**

After HSV-1 induction, the rat's escape latency was increased, the time spent on the platform in the target quadrant and the number of platform crossings significantly decreased. In addition, there was an increase in apoptosis in the hippocampus, accompanied by elevated levels of p–p38 and decreased levels of p-CREB. However, these effects could be improved by acupuncture treatment. Interestingly, SB203580 plays a similar role to acupuncture, and acupuncture could further enhance the impacts of SB203580 on cognitive function and apoptosis in hippocampus in HSE rats.

**Conclusion:**

Acupuncture improves spatial learning and memory impairment caused by HSV-1 in rats. The functional mechanism of acupuncture may be through the p38 MAPK/CREB pathway.

## Highlights

1. Acupuncture improves spatial learning and memory impairment caused by HSV-1 in rats.
2. Acupuncture regulates the activation of p38 MAPK/CREB signaling pathway in hippocampus of HSE rats.
3. Acupuncture improves HSE rats through the p38 MAPK/CREB signaling pathway.

## Introduction

Herpes simplex encephalitis (HSE) is an acute infectious disease of the central nervous system brought on by the herpes simplex virus (HSV), accounting for more than 50% of the known cases of viral encephalitis [1]. The herpes virus is a double-stranded DNA virus that spreads easily among people. More than 90% of HSE infection cases are caused by HSV-1, and the mortality rate is as high as 70% [2]. It was found that about 90% of patients suffered from a mental disorder, and 75% of patients experienced cognitive dysfunction to varying degrees 1 month after the onset of HSE [3]. This mental disorder not only has a significant impact on patients' lives but also affects work performance and social security. Previous studies have suggested that the cognitive dysfunction caused by HSE is related to HSV-1-induced neuronal apoptosis [4, 5]. This suggests that inhibiting HSV-1-induced neuronal apoptosis may be beneficial in improving the cognitive dysfunction caused by HSE.

Acupuncture has a long history of treating nervous system diseases in China. The accumulated evidence shows that acupuncture has significant therapeutic effects on various diseases associated with cognitive dysfunction, such as post-stroke cognitive impairment and Alzheimer's disease [6, 7]. Notably, Jin et al. reported a case in which they used acupuncture to treat an HSE patient who was trapped in a persistent vegetative state. After seven treatments, the patient's consciousness and intelligence were restored [8]. However, at present, the mechanism of acupuncture treatment to alleviate HSE is not clear. Previous studies have reported that acupuncture can protect brain neurons from damage and improve cognitive dysfunction [9]. Accumulated evidences have also shown that acupuncture can have a wide range of neuroprotective effects by inhibiting neuronal apoptosis [10]. Therefore, we speculate that acupuncture may improve the cognitive dysfunction caused by HSE by inhibiting HSV-1-induced apoptosis in hippocampus.

The p38 mitogen-activated protein kinase (MAPK)/cAMP-response element binding protein (CREB) pathway exerts a pivotal effect in the development and growth of neurons, and it is also a common signal transduction pathway in acupuncture treatment for nervous system diseases [11]. In addition, numerous investigations have reported that activated p38 MAPK/CREB pathway is associated with cognitive dysfunction [12]. Moreover, it seems that regulating CREB can inhibit the infection caused by HSV-1 [13].

To sum up, we speculate that acupuncture may inhibit apoptosis in hippocampus and cognitive dysfunction caused by HSE by regulating the p38 MAPK/CREB pathway. This finding could offer new insights for the treatment of HSE.

## Materials and methods

### Animals

This study was approved by the Animal Ethics Committee of The First People′s Hospital of Zunyi (Zunyi, China). Sprague–Dawley (SD) rats (*n* = 56, 6 weeks, male), weighing 200–250 g were obtained from Vital River Laboratory Animal Technology Co. Ltd. (Beijing, China). Rats were maintained in a room with a 12-h light/12-h dark cycle, 50% humidity, and a constant temperature of 21 ± 0.5 ℃.

### Preparation of virus

HSV-1 virus was prepared as described above [14]. For 48 h, the HSV-1 virus strain was grown in VERO cells (SUNNCELL, Wuhan, China). The virus titer was ascertained by collecting the supernatant after around 80% of VERO cells showed larger, rounded, increased refraction, or fused lesions.

### HSE model establishment

Following the administration of pentobarbital sodium anesthesia to the rats, a subcutaneous injection of HSV-1 virus suspension (2 × 10^6^ PFU, 100 μL) was made into the right side of the rats' beard base [15]. The survival status and behavior of rats were observed and recorded every day.

### Acupuncture

Three days after the HSE modeling, animals were treated with acupuncture without anesthesia for 30 min each time for 30 days as previously described with minor modifications [16]. During acupuncture, rats were immobilized on a plastic holder in the supine position. The specific steps are as follows: after cleaning the rat skin with 75% alcohol, the sterilized stainless steel needles (0.2 × 5 mm, Suzhou Huanqiu Acupuncture Medical Appliance Co., Ltd, China) were inserted into the indicated acupoints (*Baihui* (GV20), *Shenmen* (HT7), *Shenting* (GV24), *Xuanzhong* (GB39), and *Sanyinjiao* (SP6) [17]) to a depth of 4 mm. SP6 is 0.5 cm above the posterior end of the medial ankle, whereas GB39 is located 1.0 cm anterior to the lateral ankle [18]. GV20 is located at the intersection of the sagittal midline of the head and the midpoint of the coronary of both ears [19]. HT7 is located at the carpal crease at the edge of the ulna [20]. GV24 is located in the anterior midline to the junction of the frontoparietal bones [21]. Control rats did not receive acupuncture intervention, but were treated and immobilized in the same manner as the rats in the acupuncture group.

### Animal grouping

There were two sections to the animal tests. Rats were split into three groups for the first section: control, HSV-1, and HSV-1 + acupuncture. The rats in the control group did not receive HSV-1 infection or acupuncture. The rats in the HSV-1 group were infected with HSV-1 but did not undergo acupuncture. The rats in the HSV-1 + acupuncture group received both HSV-1 infection and acupuncture treatment. Rats were split into four groups for the second section: control, HSV-1, HSV-1 + SB203580 (MPAK inhibitor, MedChemExpress, Monmouth Junction, NJ, USA; rats that were injected with HSV-1 were given an intraperitoneal injection of 5 µg/kg of SB203580 once every day for 2 weeks), and HSV-1 + SB203580 + acupuncture (rats injected with HSV-1 received an intraperitoneal injection of SB203580 and acupuncture at GV20, HT7, GV24, GB39, and SP6 acupoints). In both the first and second sections, all groups of rats (including the control, HSV-1, and HSV-1 + SB203580 groups) received the same immobilization treatment as the acupuncture group. After treatment, water maze test was performed in each group of rats. Within 24 h after the water maze experiment, rats were sacrificed (Cervical dislocation after anaesthesia with intraperitoneal injection of sodium pentobarbital) and hippocampus were collected for molecular biological experiments.

### Morris water maze (MWM) test

The learning and memory abilities of rats were tested using the MWM as previously described [22]. MWM was carried out for 6 days after acupuncture and SB203580 treatment. MWM is a cylindrical pool measuring 150 cm × 60 cm and divided into four quadrants. In the middle of a quadrant, there was a platform measuring 9 cm × 30 cm. A camera connected to the computer system is positioned above the maze to record the movement track of rats and simultaneously determine the platform latency and other parameters. Water is injected into the pool beforehand, and black ink is added to the water to prevent the rats from seeing the platform. The water surface is 2 cm above the platform, and the water temperature is controlled at 23 ± 1 ℃. The rats were trained four times a day from the first to the fifth day. During each training session, a random water entrance site was selected, and the rats were placed in the water with their backs against the pool wall. The escape latency, which is the time it took for rats to find the platform, was noted. Rats discover the platform in less than 60 s; in such cases, the time taken to find it is recorded. The escape latency is 60 s if the rat cannot locate the platform within that time. The platform was taken down, and the rats fell into the water through the same entry point on the sixth day. The data needed for the experiment was recorded.

### Quantitative real-time PCR (qPCR) assay

Total RNA was extracted from the hippocampus of rats by Trizol reagent (Sangon Biotech, Shanghai, China) and quantified by ultraviolet spectrometry at 260 nm. Using the cDNA Synthesis Master Mix (Sangon Biotech), the RNA was reverse-transcribed into complementary DNA (cDNA). The quantification of interest genes was analyzed by qPCR, which was performed with Probe qPCR Mix (Beyotime, Shanghai, China), using a Real-Time PCR system (Thermo Fisher Scientific, Waltham, MA, USA). The internal reference was glyceraldehyde-3-phosphate dehydrogenase (GAPDH). The results were calculated by the 2^−ΔΔCt^ method. The primer sequences are listed in Table 1.

**Table 1:** Primers used in this study

Gene	Primer sequence
Bcl-2 Forward	5’-GGTGAACTGGGGGAGGATTG-3’
Bcl-2 Reverse	5’-AGAGCGATGTTGTCCACCAG-3’
Bax Forward	5’-CACGTCTGCGGGGAGTCA-3’
Bax Reverse	5’-TAGGAAAGGAGGCCATCCCA-3’
GAPDH Forward	5’-AGGAAATGATGACCTCCTGAACT-3’
GAPDH Reverse	5’-GAAGATGCGGTCACCTCACA-3’

### Western blot

The total protein was extracted from hippocampus using Tissue Protein Extraction Kit (YaJi Biological, Shanghai, China). The protein concentration was measured by BCA Protein Assay kit (Life-iLab, Shanghai, China). Equivalent amounts of proteins were separated by a sodium dodecyl sulfate–polyacrylamide gel electrophoresis (SDS–PAGE) gel (YaJi Biological), and blotted onto a polyvinylidene fluoride membrane (PVDF, Epizyme, Shanghai, China). After being blocked in the blocking buffer (Life-iLab), the membranes were incubated with the primary antibodies (Table 2). After being incubated at 4 ℃ for overnight, the membranes were incubated with secondary antibody (Table 2). The chemiluminescent signals were detected using Odyssey Infrared Imaging System (LI-COR, USA) and an ECL kit (Life-iLab).

**Table 2:** Antibodies used in this study

Antibody	Catalog	Dilution	Manufacturer
Bcl-2	ab194583	1/1000	Abcam, Cambrige, MA, USA
Bax	ab32503	1/2000	
p–p38	#9211	1/1000	Cell Signaling, Dancers, MA,USA
P38	#9212	1/1000	
P-CREB	ab32096	1/5000	Abcam
CREB	#9197	1/1000	Cell Signaling
β-Actin	ab8227	1/2000	Abcam
Goat anti rabbit	ab205718	1/10000	Abcam

### Statistical analysis

Statistical analysis was undertaken using GraphPad Prism software (Version 8.0, USA). The measurement data were shown as mean ± standard deviation (SD). Multiple groups were done through a one-way analysis of variance followed by Tukey’s post hoc test. In all statistical analysis, a level of significance of *p* < 0.05 was assumed.

## Results

### Acupuncture improves spatial learning and memory impairment caused by HSV-1 in rats

**Fig. 1:**
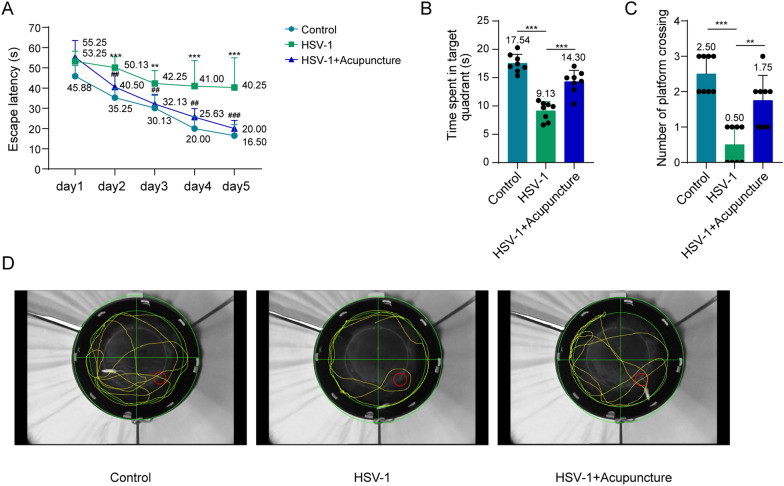
Acupuncture improves the abilities of spatial learning and memory impairment induced by HSV-1 in rats. **A** Escape latency on 1–5 day training phase. **p < 0.01, ***p < 0.001 vs. control; ##*p* < 0.01, ###*p* < 0.001 vs. HSV-1. **B** Time spent in the target quadrant during the probe trial. **C** Number of platform crossing during the probe trial. **D** Representative swimming trajectory of each group. *n* = 8. Data were displayed as mean ± SD. **p < 0.01, ***p < 0.001

After giving rats acupuncture, we utilized MWM to test their memory and learning. Throughout the first 5 days of constant training, each group of rats' escape latency dramatically decreased as training intensity increased; The HSV-1 group's escape latency grew dramatically from the second training day onwards in comparison to the control group; on the fourth and fifth training days, the HSV-1 + acupuncture group's escape latency was much lower than the HSV-1 group's (Fig. 1A). On the sixth day, comparing HSV-1 rats to the control group, the former spent noticeably shorter periods on the platform and crossed a smaller number in the target quadrant (Fig. 1B, C). However, HSV-1 rats that received acupuncture treatment spent significantly more time on the platform and crossed more numbers in the target quadrant compared to HSV-1 rats (Fig. 1B, C). Figure 1D displayed the representative swimming routes of the three groups of rats in the spatial exploration experiment on the sixth day. The above results demonstrate that acupuncture can enhance spatial learning and memory abilities of rats induced by HSV-1.

### Acupuncture inhibits apoptosis in hippocampus induced by HSV-1 in rats

**Fig. 2:**
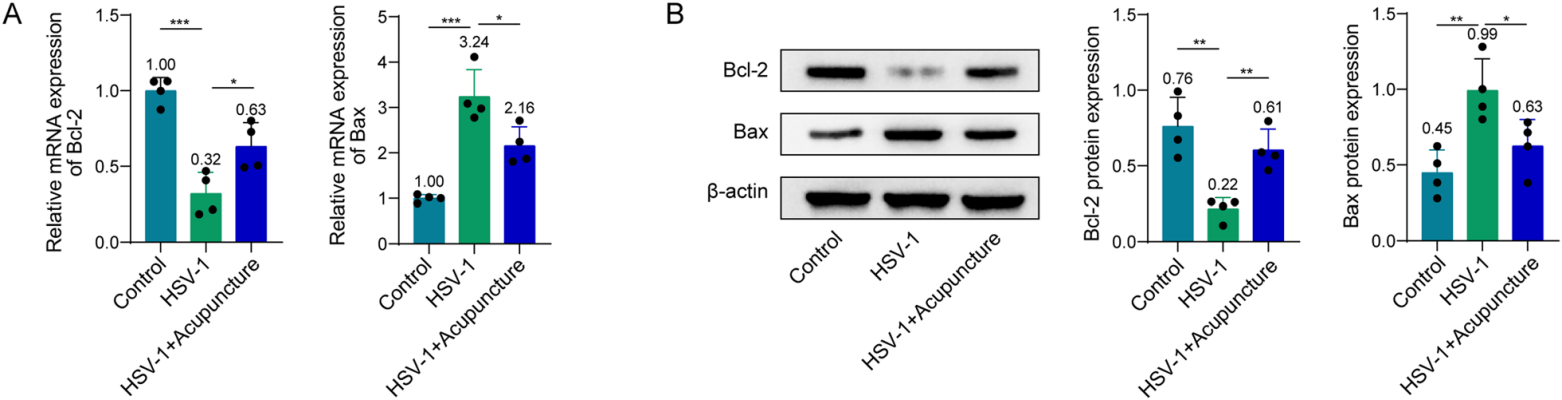
Effects of acupuncture on apoptosis in hippocampus induced by HSV-1 in rats. **A** mRNA expression of Bcl-2 and Bax in hippocampus was determined by qPCR. **B** Protein levels of Bcl-2 and Bax in hippocampus were determined by western blot. *n* = 4. Data were displayed as mean ± SD. *p < 0.05, **p < 0.01, ***p < 0.001

In the rat hippocampus, we found that genes and proteins linked to apoptosis were expressed. The findings demonstrated that whilst HSV-1 considerably decreased the mRNA and protein levels of Bcl-2, it significantly raised those of Bax. However, these effects of HSV-1 were partially reversed by acupuncture (Fig. 2A, B). According to the aforementioned findings, acupuncture may inhibit the apoptosis in hippocampus induced by HSV-1 in rats.

### Acupuncture regulates the activation of p38 MAPK/CREB signaling pathway in hippocampus of HSV-1 rats

**Fig. 3:**
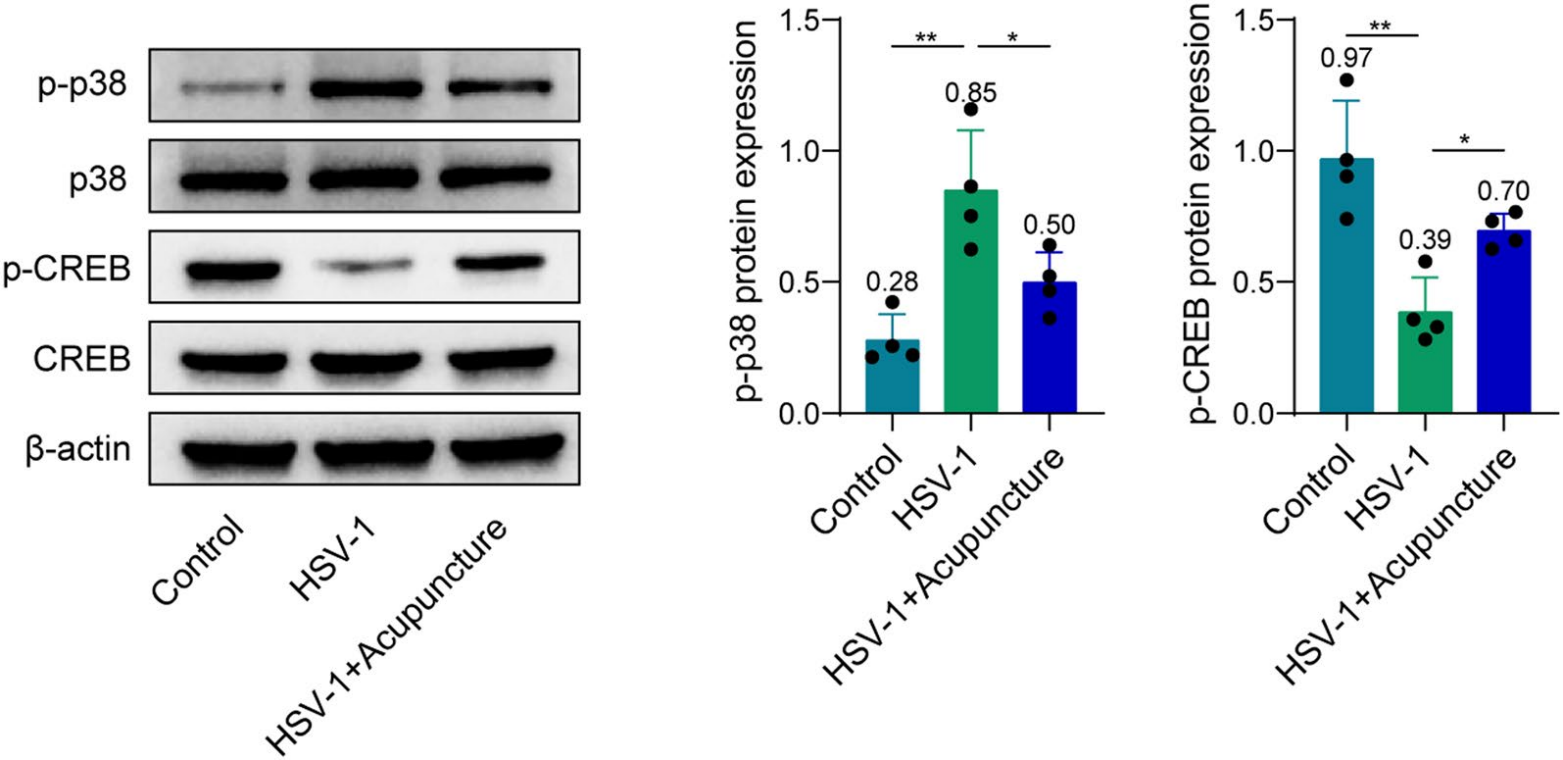
Effects of acupuncture on activation of p38 MAPK/CREB pathway induced by HSV-1. The protein levels of p–p38, p38, p-CREB, and CREB in hippocampus were determined by western blot. *n* = 4. Data were displayed as mean ± SD. *p < 0.05, **p < 0.01

In view of the important role of p38 MAPK/CREB pathway in cognitive function, we detected the expressions of proteins related to p38 MAPK/CREB pathway. The findings demonstrated that HSV-1 infection caused the increase of p–p38 level and the decrease of p-CREB level in hippocampus of rats (Fig. 3). However, acupuncture can partially recover the abnormal levels of p–p38 and p-CREB caused by HSV-1 (Fig. 3). These findings demonstrate that acupuncture inhibits the p38 MAPK/CREB signaling pathway in hippocampus of HSV-1 rats.

### Acupuncture inhibits apoptosis in hippocampus induced by HSV-1 in rats through the p38 MAPK/CREB pathway

**Fig. 4:**
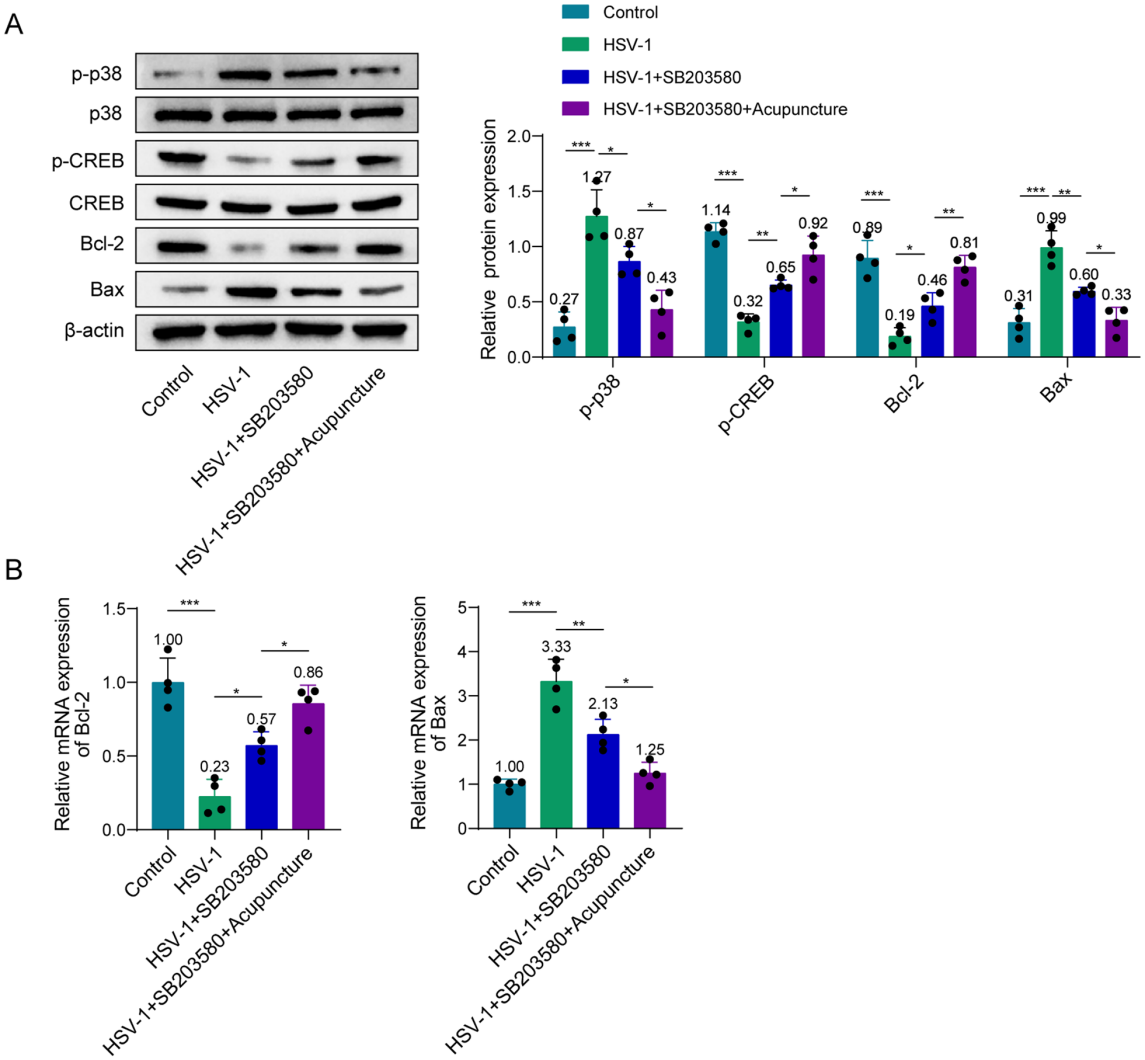
Acupuncture inhibits apoptosis in hippocampus through the p38 MAPK/CREB pathway. **A** Protein levels of p–p38, p38, p-CREB, CREB, Bcl-2, and Bax in hippocampus were determined by western blot. **B** mRNA expression of Bcl-2 and Bax in hippocampus was determined by qPCR. *n* = 4. Data were displayed as mean ± SD. *p < 0.05, **p < 0.01, ***p < 0.001

To confirm if the p38 MAPK pathway is involved in the effect of acupuncture on improving the cognitive function of HSV-1 rats, we used p38 MAPK inhibitor SB203580. After intervention with SB203580, the regulation of HSV-1 on apoptosis-related proteins, p–p38 and p-CREB was partly reversed (Fig. 4). Interestingly, acupuncture can further amplify the effects of SB203580 (Fig. 4). These findings suggest that acupuncture inhibits apoptosis in hippocampus through the p38 MAPK/CREB pathway.

### Acupuncture improves spatial learning and memory impairment induced by HSV-1 in rats through the p38 MAPK/CREB pathway

**Fig. 5:**
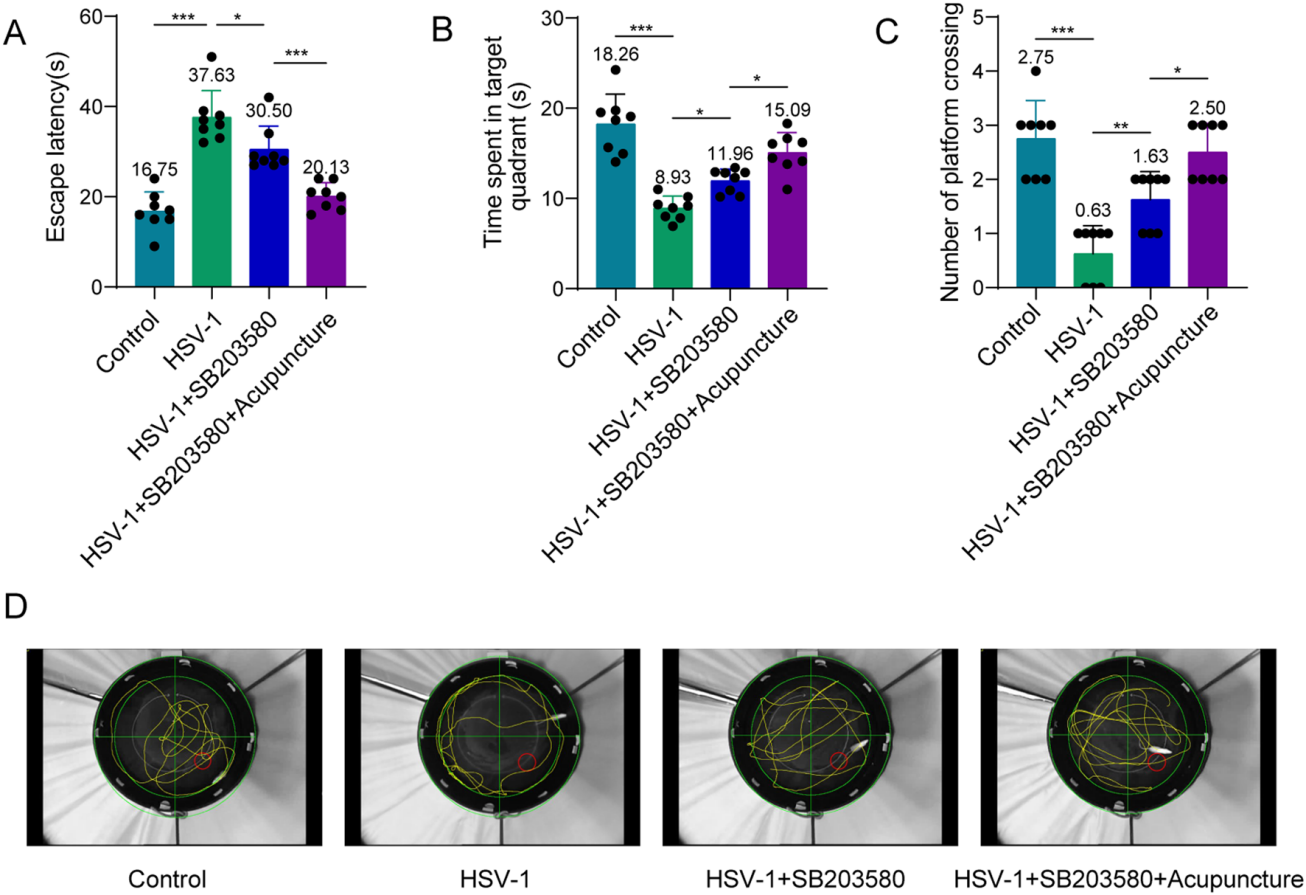
Acupuncture improves spatial learning and memory abilities of rats induced by HSV-1 through the p38 MAPK/CREB pathway. **A** Escape latency on day 5. **B** Time spent in the target quadrant during the probe trial. **C** Number of platform crossing during the probe trial. **D** Representative swimming trajectory of each group. *n* = 8. Data were displayed as mean ± SD. *p < 0.05, **p < 0.01, ***p < 0.001

In MWM experiment, compared with HSV-1 group, SB203580 shortened the escape latency, increased the platform spent time and the numbers of crossing the platform (Fig. 5A–C). Interestingly, acupuncture can further amplify the effects of SB203580 (Fig. 5A–C). Figure 5D displayed the representative swimming routes of the four groups of rats in the space exploration experiment after the platform was removed on the sixth day. The above results indicate that acupuncture improves spatial learning and memory impairment caused by HSV-1 in rats through the p38 MAPK/CREB pathway.

## Discussion

Acupuncture is widely used to treat various forms of cognitive dysfunction in clinics, and it has shown good clinical effects [23]. In clinical practice, acupuncture practitioners typically use combined acupoints because combining acupoints in acupuncture can generate synergistic reactions that enhance the clinical therapeutic effect [24, 25]. GV20, HT7, GV24, GB39, and SP6, these five acupoints have been proven to be effective in treating various forms of cognitive impairment [26, 27]. Here, we selected these five acupoints for acupuncture to investigate the impact of acupuncture on HSE-induced dysfunction [28].

The learning, memory, and neuroplasticity of rodents and humans largely depend on the hippocampus. The ability of learning and spatial memory in rats induced by HSV-1 declined dramatically, and the apoptosis in hippocampus boosted. According to the principles of Traditional Chinese Medicine (TCM) and clinical experience, the acupoints associated with cognitive impairment are centered on 11 meridians (GV, CV, BL, LR, GB, KI, ST, SP, HT, PC, and TE) in the head and distal limbs [29]. In addition, acupuncture points may be excitable muscle/skin–nerve complexes containing a high density of nerve endings, corresponding to the fact that stimulation of these points can send signals to the spinal cord [30]. Previous studies have reported that acupuncture at the HT7, GV20, and SP6 acupoints can improve spatial learning and memory impairment in rats and reduce neuronal apoptosis [27]. Acupuncture at the GB39 acupoint can improve the neurological and motor functions of patients [31]. Acupuncture at the GV24 and GV20 acupoints can improve cognitive impairment after a stroke [32]. In addition, the study found that acupuncture at the GV24 acupoint can alleviate the learning and memory impairment of rats through the serotonin pathway in the hippocampus [33]. Consistently, we found that the cognitive dysfunction and apoptosis in hippocampus induced by HSV-1 were improved to some extent after acupuncture at 5 acupoints.

It has been demonstrated that the MAPK family, which includes the p38 MAPK kinase, is activated following stimulation by viruses or virus-related proteins. Therefore, this pathway is crucial for the fate of virus-infected cells [34]. In previous studies, it was reported that HSV-1 could induce the activation of the p38 MAPK pathway [35]. Consistently, our results also show that after HSV-1 induction, the phosphorylation level of p38 in the rat hippocampus increases. On the other hand, CREB is very important for learning and long-term memory. It is found that upregulating CREB activity can improve memory deficits [36]. This may be because CREB activation can increase the expression of downstream neuroprotective genes, such as BDNF, thus protecting neurons from apoptosis [37]. In our study, we found that HSV-1 induction can decrease the phosphorylation level of CREB, suggesting that HSV-1 may exacerbate cognitive dysfunction by inhibiting CREB activity. Our results also showed that the use of SB203580 can promote the phosphorylation of CREB, inhibit apoptosis in hippocampus, and improve the learning and spatial memory impairment induced by HSV-1 in rats. Consistently, according to the prior research, SB203580 could raise the level of CREB phosphorylation in the hippocampus of mice with Alzheimer's disease, thereby improving the cognitive dysfunction in mice [38]. Notably, acupuncture reversed the upregulation in p–p38 levels and the decline in p-CREB levels mediated by HSV-1, and amplified the effects of SB203580. Consistently, in the previous studies, electroacupuncture at GV20, GV24 and *Zusanli* (ST36) improved the cognitive dysfunction of rats by inhibiting p38 MAPK pathway [39], and electroacupuncture at GV20 and HT7 improved the cognitive dysfunction of rats by increasing CREB activity in hippocampus [40]. In addition, acupuncture at GB39 is reported to attenuate neuronal cell death in a model of focal ischemic middle cerebral artery occlusion [41]. Electroacupuncture stimulation of SP6 inhibits the p38 MAPK pathway, thereby attenuating apoptotic index in hippocampal cells of cerebral ischemia/reperfusion-injured rats [42]. This means that acupuncture may inhibit apoptosis of hippocampus induced by HSV-1 by regulating p38 MAPK/CREB pathway, thus improving cognitive dysfunction in rats. In summary, our results indicate that acupuncture may inhibit apoptosis in hippocampus and cognitive dysfunction caused by HSE by regulating P38 MAPK/CREB pathway. This further emphasizes the importance of acupuncture in the treatment of cognitive dysfunction. This study will provide new ideas for the treatment of HSE.

## Data Availability

The data sets used or analyzed during the current study are available from the corresponding author on reasonable request.

## Abbreviations

HSE: Herpes simplex encephalitis
HSV-1: Herpes simplex virus type-1
BDNF: Brain derived neurotrophic factor
qPCR: Quantitative real-time PCR
MWM: Morris water maze
GAPDH: Glyceraldehyde-3-phosphate dehydrogenase
SD: Standard deviation
MAPK: Mitogen-activated protein kinase
CREB: CAMP-response element binding protein
